# Genótipo da Paraoxonase-1 e Angiograma Positivo para Doença Arterial Coronariana

**DOI:** 10.36660/abc.20220665

**Published:** 2023-03-27

**Authors:** Rujittika Mungmunpuntipantip, Viroj Wiwanitkit

**Affiliations:** 1 Consultor Acadêmico Privado Bangkok Tailândia Consultor Acadêmico Privado, Bangkok – Tailândia; 2 Universidade Dr. DY Patil Pune Índia Universidade Dr. DY Patil, Pune – Índia

**Keywords:** Doença Arterial Coronariana/genética, Mortalidade, Polimorfismo Genético, Predisposição Genética para Doença, Fatores de Risco

**Prezado Editor,**

Gostaríamos de compartilhar ideias sobre a publicação “Associação do Genótipo e Fenótipo da Paraoxonase-1 com Angiograma Positivo para Doença Arterial Coronariana.”^1^ Soflaei et al.,^1^ observaram que a presença do alelo G do polimorfismo de nucleotídeo único rs662 está independentemente associada a um risco aumentado de doença arterial coronariana (DAC).^1^ O efeito do polimorfismo é investigado neste estudo. A DAC pode ou não ser afetada pelo componente hereditário estudado neste relatório. Ambos concordamos que a DAC pode estar relacionada ao componente genético subjacente sob pesquisa. No entanto, a DAC tem sido associada a inúmeras variações genéticas. Exemplos de polimorfismos de genes incluem o efeito de polimorfismos (M235T e T174M) no gene do angiotensinogênio (AGT), TGFB1 rs1800469 e BCMO1 rs6564851.^2-3^ Pesquisas futuras devem examinar as implicações de variantes genéticas inesperadas e possivelmente desconcertantes.
