# Supplementary material for: Genótipo da Paraoxonase-1 e Angiograma Positivo para Doença Arterial Coronariana
Source: Arq Bras Cardiol. 2023 Mar 27;120(3):e20220665. [Article in Portuguese] doi: 10.36660/abc.20220665 (PMC10263450; doi:10.36660/abc.20220665)
Supplement: Translation

# Translation: Paraoxonase-1 Genotype and Angiogram Positive Coronary Artery Disease

**Authors:** Rujittika Mungmunpuntipantip, Viroj Wiwanitkit

**Affiliations:** 1 Bangkok Thailand Private academic consultant, Bangkok – Thailand; 2 Pune India Universidade Dr. DY Patil, Pune – India

**Keywords:** Coronary Artery Disease/genetics, Mortality, Polymorphism, Genetic, Genetic Predisposition to Disease, Risk Factors

**Dear Editor,**

We would like to share ideas on the publication “Association of Paraoxonase-1 Genotype and Phenotype with Angiogram Positive Coronary Artery Disease”. ^1^ Soflaei et al.^1^ noted that The presence of the G allele of the rs662 single nucleotide polymorphism is independently associated with an increased risk of coronary artery disease (CAD).^1^ The effect of polymorphism is investigated in this study. CAD might or might not be impacted by the hereditary component studied in this report. We both concur that CAD may be related to the underlying genetic component under research. However, CAD has been linked to numerous genetic variations. Examples of gene polymorphisms include the effect of polymorphisms (M235T and T174M) on the angiotensinogen gene (AGT), TGFB1 rs1800469, and BCMO1 rs6564851.^2-3^ Future research should examine the implications of unexpected, possibly perplexing genetic variants.
